# In vitro digestibility of hydrothermally modified *Bambara* groundnut (*Vigna subterranean* L.) starch and flour

**DOI:** 10.1002/fsn3.510

**Published:** 2017-11-24

**Authors:** T. Adeniyi Afolabi, Amarachi O. Opara, Sharafadeen O. Kareem, Fatai O. Oladoyinbo

**Affiliations:** ^1^ Department of Chemistry Federal University of Agriculture Abeokuta Nigeria; ^2^ Department of Microbiology Federal University of Agriculture Abeokuta Nigeria

**Keywords:** Annealing, bambara groundnut, digestibility, heat‐moisture treatment, resistant starch

## Abstract

In‐vitro digestibility and functional properties of Bambara groundnut (*Vigna subterranean*) (BG) flour, and its native and hydrothermally modified starches were investigated. The isolated native starch (BNS) was modified by annealing at 50°C for 48 hr (BAS), and heat‐moisture treated at 100°C for 16 hr at 25% moisture level (BHS). The crude protein of BG flour was 16.88%. The amylose content of the flour and native starch was 6.10% and 27.70%, respectively. Hydrothermal modification increased the gelatinization profile of the starch but reduces its pasting properties. Swelling and solubility of the flour and starches increased with increase in temperature. X‐ray diffraction revealed BNS and BAS exhibited “Type C” crystallinity pattern while BHS was “Type A.” The BNS granule was oval, its diameter between 22 and 30 μm, with no significant change in the morphology of BAS and BHS. The BG flour had 33% resistant starch and 11.63% digestible starch. Heat‐moisture treatment enhanced the resistant starch content of the native starch significantly.

## 1 INTRODUCTION

The number of plant species which nourish humanity is remarkably limited; less than 300 plants species out of 195,000 edible plants are used for food (Simopoulos, 1999). Approximately 17 plant species provide 90% of mankind's food supply, with cereal grains having the greatest percentage (Teixeira et al., 2016). There is need to explore and study other lesser known nutritious crops such as legumes used for food. One of such is Bambara groundnut (BG), an underutilized and lesser known legume, highly covet for its starch and protein. BG is rich in carbohydrates, proteins, and lipid (Baoua, Amadou, Baributs, & Murdock, 2014; Murevanhema & Jideani, 2013), similar to other popular legumes such as cowpea, soyabean, pea etc. (Wang & Castonguay, 2014). BG is also a good source of calcium, fiber, potassium and iron and has high essential amino acids content (De‐Kock, 2004). These attributes make BG one of the nutritious food generally consumed by the populace, most especially the rural dwellers with little opportunity to obtain protein‐rich food sources. BG pods are eaten as a fresh nut, boiled after drying or grounded into flour. BG flour has a variety of uses in the confectionary industry and also for medicinal purposes. BG are boiled and salted, eaten as a snack, or roasted (Murevanhema & Jideani, 2013). In Nigeria, BG is processed into bean cake, and its flour is widely used in compounding infant food supplements. Its starch is also extracted and used in the preparation of local delicacies.

The development of a value‐added product from starch depends on a thorough knowledge of its structure and functional properties (Piyarat, 2008). Due to native starch inherent poor mechanical properties and high sensitivity to moisture, it can be modified by physical, enzymatic, and chemical modifications to produce functional starches with special properties. The effects of physical modification via hydrothermal modification on starch (such as annealing and heat – moisture treatment) are widely reported in the literature (Jacobs & Delcour, 1998; Kim & Huber, 2013; Ovando‐Martinez, Whitney, Reuhs, Doehlert, & Simsek, 2013; Wang, Wang, Wang, & Wang, 2017).

Generally, starch consumed by humans can be categorized into three different groups’ namely digestible starch, partially digested starch and resistant starch (Englyst & Cummings, 1987). *Digestible starch* is fully digested in the small intestine; *partially digested starch* is digested in the small intestine but not completely, some amount escape digestion; while *resistant starch* completely withstands digestion in the small intestine of a healthy human being. As a consequence of resistant starch's positive impact on health especially in obesity control (Shen, Zhang, Dong, Ren, & Chen, 2015), resistant starch has drawn considerable attention in food formulations with health benefits. Obesity and diabetes have become major public health concerns worldwide, and the number of cases is increasing exponentially every year (Higgins, 2014). Therefore, the expedient solution to this may be the development of foods with high resistant starch, that slow down the rate of digestion of glucose from ingested carbohydrate sources which helps to blunt glycemia, reduces insulin requirements and causes satiety (Miao, Jiang, Cui, Zhang, & Jin, 2015). The health benefit of resistant starch to its consumers also include decreasing the risk of colorectal cancer, lowering plasma cholesterol and triglyceride concentrations, enhancing vitamin and mineral absorption etc. (Aravind, Sissons, Fellows, Blazek, & Gilbert, 2013).

The worldwide increase in Type II diabetes (Whiting, Guariguata, Weil, & Shaw, 2011) has triggered increased interest in the use of legumes for the production of functional foods with low glycemic index (Piecyk, Druzynska, Worobiej, Wolosiak, & Ostrowska‐Ligeza, 2013). Due to its growing demand as the bean of choice in infant food formulation (Afolabi, 2012), especially among the rural poor in Nigeria and West Africa, there is need to understand BG digestibility. Previous studies on BG flour and starch had focused on its physical properties, chemical and physical modifications, breeding, genotype, applications, etc. (Adebowale & Lawal, 2002; Adebowale & Lawal, 2004; Afolabi, 2012; Eknayake, Jansz, & Nair, 1999; Kaptso et al., 2015; Murevanhema & Jideani, 2013; Ogundele, Minaar, & Emmambux, 2017; Oyeyinka, Singh, Ma, & Amonsou, 2016; Oyeyinka, Singh, Patrick, Gerrano, & Amonsou, 2015; Uarrota et al., 2013). Ademiluyi, Oboh, Boligon, and Athayde (2015) also reported the health benefit of fermented BG in diabetes treatment. However, there is a paucity of information on BG digestibility, the objective of this study, therefore, is to evaluate the in vitro digestibility and functional properties of BG flour and its starches. It is hoped that data generated from this study will enhance the starch's industrial application most especially in the food and pharmaceutical industries.

## 2 MATERIALS AND METHODS

### 2.1 Starch isolation

BG (*Vigna subterranean*) was purchased at Bodija market, Ibadan, Oyo state, Nigeria, and the bean was screened to eliminate defective seeds. BG bean was milled to produce the Bambara groundnut flour (BGF) used in the study. Its starch was isolated as described by Afolabi (2012).

### 2.2 Hydrothermal modification

The native starch of BG starch (BNS) was hydrothermally modified by heat‐moisture treatment at 100°C for 16 hr at 25% moisture level (BHS), while annealing was carried out at 50°C for 48 hr (BAS) following the method of Adebowale, Afolabi, and Olu‐Owolabi (2005). The purity of the isolated starch was evaluated by determining their protein, fat, and ash content.

### 2.3 Proximate composition

The AOAC International (2005) method was used in determining the ash, moisture, fat, crude fiber, and protein contents of the starch sample. The carbohydrate content was determined by difference. The AACC (2003) method was employed for the determination of the amylose content of the starch and flour sample.

### 2.4 Swelling power and starch solubility

The effect of temperature and pH on solubility and swelling power of the starch samples were investigated following the methods of Afolabi et al. (2012).

### 2.5 Pasting properties

A Rapid Visco‐Analyser (RVA Tecmaster, Perten instrument, Sweden) was used to determine the pasting properties of the starch using AACC (2003) method.

### 2.6 Thermal properties

The gelatinization parameter of the native and modified BG starch was studied with differential scanning calorimeter (DSC) using the procedure of Afolabi et al. (2012).

### 2.7 X‐ ray diffraction

The crystallinity of the native and modified starch was determined with a Rigaku D‐Max‐ 2200 X‐ray diffractometer (Rigaku Denki Co. Tokyo, Japan). The scanning region of the diffraction angle was from 3 to 40°, with target voltage 40 KV, target current, 100 mA, and aging time 5 min. The relative crystallinity of the starches was determined as enumerated by Afolabi (2012).

### 2.8 Granule morphology

Granule morphology of the starch was studied by scanning electron microscope, SEM (Hitachi TM‐1000 Table‐top Scanning electron microscope) at 500 magnification.

### 2.9 Preparation of α – amylase, and glucoamylase

Alpha‐amylase and glucoamylase were produced on a solid state fermentation medium containing rice bran, soyabean flour and cassava starch mixed in ratio (10:3:1 w/w) according to Akpan and Adelaja (2004). The mixture in 250 ml Erlenmeyer flask was moistened with sterile distilled water to 60% moisture content. The medium pH was adjusted to 5.0 with 0.1 mol/L HCl, and sterilized at 121°C for 15 min. The sterilized medium was inoculated with spores of *Aspergillus niger* for α – amylase production, while spores of *Rhizopus oligoshporus* were used for glucoamylase synthesis. Both media were incubated at 30°C for 72 hr.

Crude enzyme extracts were recovered by mixing moldy bran with 0.2 mol/L acetate buffer (pH 6.0 for α–amylase, pH 4.5 for glucoamylase) in the ratio 1:4 (w/v) in conical flasks. The mixtures were then shaken on an orbital shaker at 150 rpm at 28°C for 1 hr. The extracts were then filtered using muslin cloth. The filtrates were partially purified using 70% ammonium sulfate and kept at 4°C for further use.

### 2.10 Resistant starch determination

The in vitro determination of resistant starch content of the BG flour and starches were analyzed by previously described methods (Champ, Martin, Noah, & Gratas, 1999; Englyst, Wiggins, & Cummings, 1982) with some modification. The sample (100 mg starch or flour) was mixed with sodium acetate buffer containing α‐amylase, and incubated at 37°C for 16 hr. Absolute ethanol (40 ml) was added to the mixture, equilibrated for 1 hr and centrifuged (Centrifuge 5702R Eppendorf AG 22331 Hamburg, Germany) at 3,913 *g* for 30 min. The residue was washed twice with 80% ethanol and dried at 60°C. Water (1.56 ml) and 4.0 mol/L KOH (1.5 ml) was added to the dried residue and mixed for 30 min at room temperature. To 1.5 ml of the dispersion; 12 ml of water, 0.65 ml of 2 mol/L acetic acid, and 0.1 ml of amyloglucosidase was added and shaken with the aid of a thermostated shaker (Uniscope SM101 shaking water bath, Surgifriend Medicals, England) for 90 min at 65°C.

The glucose content of the slurry was determined with glucose oxidase assay kit (Cypress Diagnostic, HB009; G‐544, Belgium), measuring the absorbance wavelength (PG instruments, T60‐U UV‐visible spectrophotometer, USA) at 505 nm. The resistant starch content was calculated as *mg of glucose* × *0.9*.

### 2.11 Total and digestible starch determination

The method of Goni, Alonso, and Saura‐ Calixto (1997) was employed in determining the total starch content of the BG flour and starches. The sample (50 mg) was dispersed in 6.0 ml of 2 mol/L KOH, and incubated for 30 min at room temperature. The solubilized starch was hydrolyzed by adding 60 μl of amyloglucosidase, then incubated at 60°C for 45 min in a thermostated shaker (Uniscope SM101 shaking water bath, Surgifriend Medicals, England), and subsequently centrifuged (Centrifuge 5702R Eppendorf AG 22331 Hamburg, Germany) at 3,170 *g*, 15 min. Glucose oxidase‐peroxidase kit (Cypress Diagnostic, HB009; G‐544, Belgium) was used to measure the glucose content of the supernatant, and the total starch content was calculated as mg of glucose* × 0.9*.

The digestible starch was determined by calculating the difference between total starch and resistant starch of the sample on dry weight basis.

### 2.12 Statistical Analysis

All determinations were carried out in triplicate and result reported as the mean ± standard deviation. The swelling and solubility profile were subjected to one‐way analysis of variance (ANOVA) using SPSS statistical software (version 20) to investigate the effect of pH and temperature on starch samples. The *Shapiro‐Wilk test* of normality and the *Levene's tests* of homogenous variance were carried out to assess the assumptions of ANOVA in order to validate the results.

## 3 RESULT AND DISCUSSION

### 3.1 Starch composition

The starch yield of BG on flour basis was 41% (Table 1), this is comparable with 40.35% (Afolabi, 2012), but higher than 37.50% (Adebowale et al., 2002) reported for BG. The discrepancy in the yield is probably due to an improved method of isolation of the starch. The BG starch yield is within the 18%–49% range reported for different pulses (Hoover, Hughes, Chung, & Liu, 2010; Mensah, 2011). The moisture content of 9.15% for BGF is at par with the 9.50% and 9.70% reported for BGF by Adebowale and Lawal (2002) and Enwere and Hung (1996), respectively. The moisture content of the native starch (BNS) is 14.11%, while hydrothermal modification reduces moisture content, probably because hydrothermal modification limit the amount of water‐retainable by starch (Zavareze & Guerra Dias, 2011).

**Table 1:** Proximate composition of bambara groundnut: flour (BGF), native starch (BNS), annealed starch (BAS), and heat‐moisture treated starch (BHS)

Parameters (%)	BGF	BNS	BAS	BHS
Moisture content	9.15 ± 0.01^b^	14.11 ± 0.25^a^	8.27 ± 0.01^c^	8.83 ± 0.15^c^
Protein	16.88 ± 0.01^a^	1.77 ± 0.00^b^	1.51 ± 0.01^d^	1.67 ± 0.01^c^
Fat content	6.98 ± 0.04^a^	2.59 ± 0.01^b^	1.51 ± 0.01^d^	1.77 ± 0.01^c^
Crude fiber	6.41 ± 0.01^a^	2.21 ± 0.01^b^	1.92 ± 0.15^c^	1.70 ± 0.01^d^
Ash	2.92 ± 0.096^a^	0.21 ± 0.15^b^	0.14 ± 0.01^b^	0.14 ± 0.05^b^
Carbohydrate	55.66	79.11	86.65	85.89
Amylose content	6.10 ± 0.04^b^	27.70 ± 0.05^a^	‐	‐
Starch yield	‐	41.00 ± 0.12^c,^ a	94.50 ± 0.11^b,^ b	98.20 ± 0.08^a,^ b

Results are means of triplicate determinations ± standard deviation. Means followed by different superscript in the same row are significantly different (*p* < 0.05).

a On flour basis.

b On native starch basis.

The 16.88% protein content of BGF (Table 1) is similar to the 16.60% reported by Enwere and Hung (1996), but higher than the 15.48% reported by Piyarat (2008) for BG flour. Adebowale and Lawal (2002), Abiodun and Adepeju (2011), and Eltayeb, Ali, Abou‐Arab, and Abu‐Salem (2011) reported higher protein content of 17.70%, 20.7%, and 22.50%, respectively for BGF. The difference in the reported values may be attributed to differences in the varieties of seeds studied. The 6.98% fat content of BG flour in this study is comparable to the 6.56% reported by Eltayeb et al., 2011; but lower than the 7.90% and 16.60% for BGF reported by Piyarat (2008) and Enwere and Hung (1996), respectively. The amylose content of the native starch, BNS was 27.7%. Oyeyinka et al. (2015) reported varied amylose contents (20–35%) among five genotypes of BG starches. High amylose content in starch has been reported to inhibit swelling during gelatinization and also increase the viscosity (Tester & Morrison, 1990).

### 3.2 Swelling and solubility

**Figure 1:**
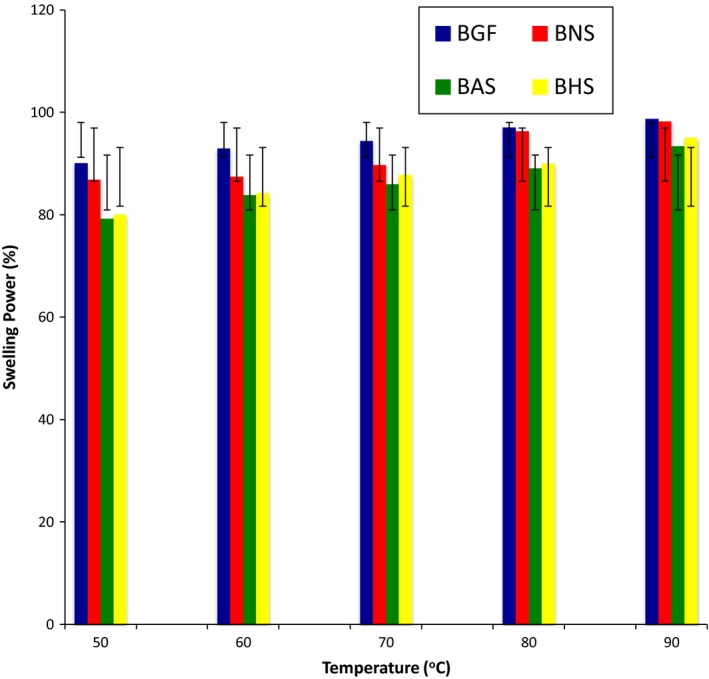
The swelling power of the bambara groundnut flour (BGF), native starch (BNS), annealed starch (BAS), and heat‐moisture treated starch (BHS) at different temperatures

**Figure 2:**
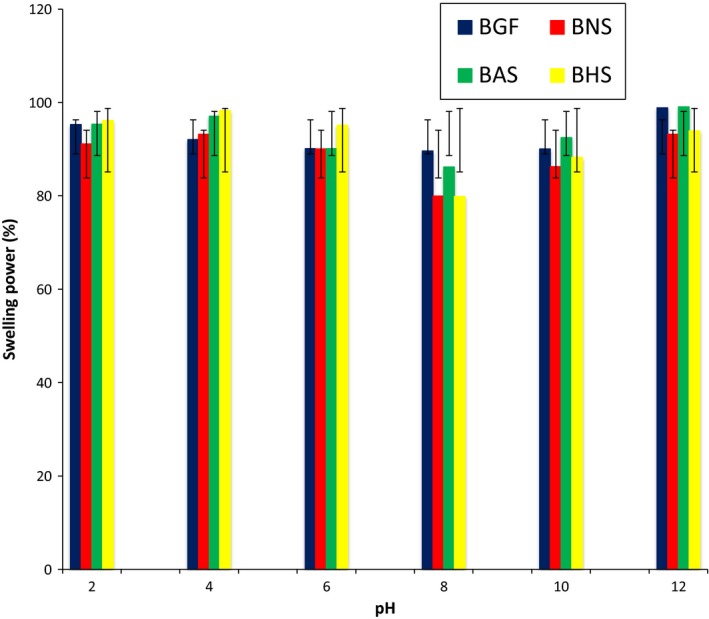
The swelling power of the bambara groundnut flour (BGF), native starch (BNS), annealed starch (BAS), and heat‐moisture treated starch (BHS) at different pH

The effect of temperature on swelling power revealed that as the temperature increased, the swelling power of the flour and starch increased (Figure 1). The increase in swelling power of the starches as the temperature increased is consistent with other reports on leguminous starches (Afolabi, 2012). Although all the starches swelled as the temperature increased, hydrothermal modification induced a reduction in the swelling power of the BG starches, with BHS having the lowest values. The reduction in the swelling power of BHS could be attributed to the increase in crystallinity and strengthening of intermolecular bonds due to heat‐moisture treatment (Singh, Chang, Lin, Singh, & Singh, 2011), which leads to a restriction in the swelling of the starch granules. Due to the inhibitory effect of amylose to swelling, starch with low total amylose contents (being less rigid) swell freely when heated (Singh, Kaur, & McCarthy, 2007). Also, the reduction in the swelling power of BG starch after annealing (BAS) could be the resultant effect that annealing induced the interaction between the degree of crystalline perfection and amylose‐amylose or amylose‐amylopectin interplay; this interaction decrease the hydration of amorphous regions of starch, thereby decreasing granular swelling of starch (Zavareze & Guerra Dias, 2011). The swelling power of the flour and starches at different pH (Figure 2) revealed that the swelling power of the starches peaked at pH 4 in the acidic medium, and at pH 12 in the alkaline medium. BHS had the highest swelling power in the acidic medium (pH 2–6), while BAS had the highest swelling power in alkaline medium (pH 8–12).

**Figure 3:**
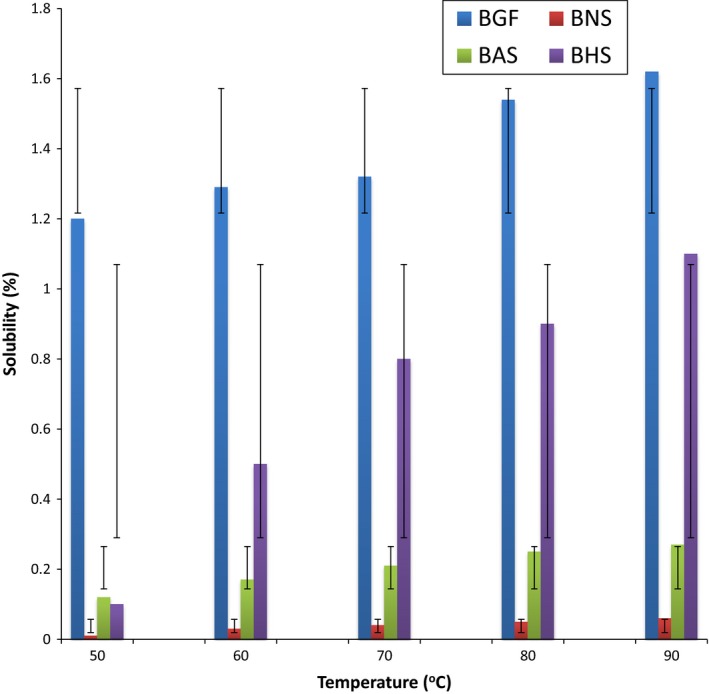
The solubility profile of bambara groundnut; flour (BGF), native starch (BNS), annealed starch (BAS), and heat‐moisture treated starch (BHS) at different temperatures

**Figure 4:**
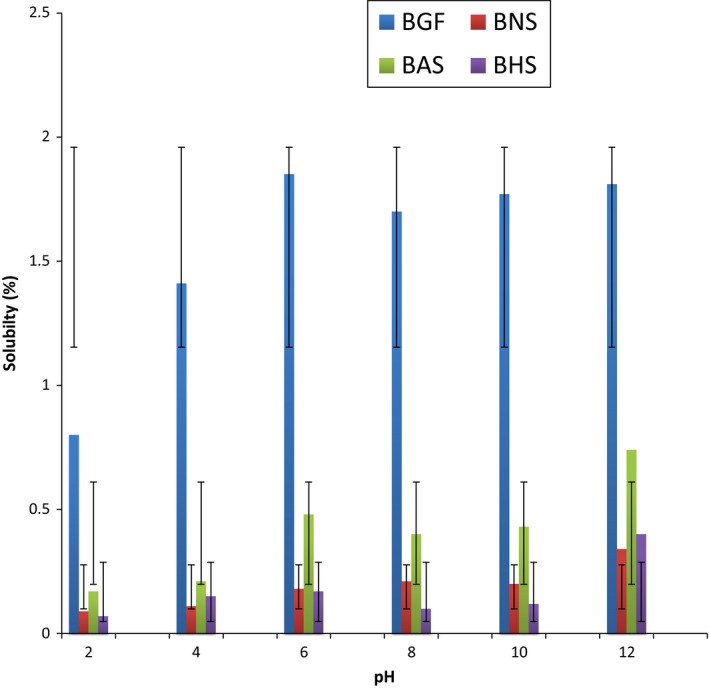
The solubility profile of bambara groundnut; flour (BGF), native starch (BNS), annealed starch (BAS), and heat‐moisture treated starch (BHS) at different pH

The solubility of BG flour and starches increased as the temperature increased (Figure 3). The observed increase in starch solubility as the temperature increased is in agreement with other reports in the literature (Afolabi, 2012). Hydrothermal modifications significantly enhance the solubility of the BG starch as the temperature increased, with BAS having the highest solubility. Starch solubility is a product of amylose leakages, which disseminates from the starch granules (Zavareze & Guerra Dias, 2011), therefore hydrothermal modification favor amylose leakages, with the concomitant increase in the solubility of the starches. The solubility of the BG samples at different pH (Figure 4) increased as the pH increased from 2 to 12, with the BG flour having the highest solubility at all pH. This observation is similar to that reported for red sorghum by Adebowale et al. (2005). The solubility of the starches in the alkaline medium (8–12) was higher than that in the acidic medium (pH 2–6). The increased solubility in the alkaline medium could be attributed to the enhanced water affinity of the starch at the alkaline pH, and partial gelatinization which usually occurs at this pH (Lawal & Adebowale, 2005). The higher swelling and solubility profile of BGF at all temperatures and pH could be attributed to the solubilization of the protein.

The statistical analysis of variance (ANOVA) between BNS, BAS, and BHS at different temperature and pH levels showed that the *Wilk Statistic* for the BNS, BAS, and BHS starch samples are 0.825, 0.815 and 0.837, respectively and their significance were all greater than 0.05. The *Levene test* has a value of 2.169 with a significance value of 1.20 for swelling index and 2.885 with a significance value of 1.662 for solubility. With respect to swelling index, there was a statistically significant difference between the three starch samples as indicated by one‐way ANOVA (*F* 4, 40) = 36.828, *p* = .000. A *Tukey post hoc test* revealed that the mean of the BNS starch sample is significantly higher than the other 2 starches – BAS and BHS and there is a statistically significant difference between the starch samples at 80°C and 90°C only, while there was no statistical difference between the three starch sample's solubility at a different temperature.

### 3.3 Pasting and thermal properties

The pasting and thermal properties of the BG starches are presented in Table 2. Hydrothermal modification significantly reduced the peak, trough, breakdown, setback, and final viscosity of the BG starch compared with its native starch in the following order: BNS > BAS > BHS (Table 2), this is similar to the report for other starches (Puncha‐Arnon & Uttapap, 2013). The low peak and final viscosity observed in BHS is similar to that reported by Hoover and Ratnayake (2002) for heat‐moisture treated pulse's starch. Pasting of starch usually occurred after gelatinization during the dissolution of starch (Qin‐lu et al., 2011). The reduced viscosity with increased pasting temperature observed after hydrothermal modification of the BG starch is consistent with that reported for other starches and attributed to structural rearrangement and starch–chain associations (Puncha‐Arnon & Uttapap, 2013).

**Table 2:** The pasting and gelatinization properties of Bambara groundnut native (BNS), annealed (BAS), and heat‐moisture treated (BHS) starch

Parameters	BNS	BAS	BHS
Pasting			
Peak viscosity, PV (RVU)	6,095 ± 0.21^a^	5,433 ± 0.83^b^	1,461 ± 0.82^c^
Trough viscosity, TV (RVU)	1,843 ± 0.14^a^	1,508 ± 0.28^b^	1,235 ± 1.11^c^
Breakdown, (PV–TV) (RVU)	4,252 ± 0.69^a^	3,925 ± 0.11^b^	226 ± 0.32^c^
Final viscosity, FV (RVU)	5,496 ± 0.15^a^	4,677 ± 0.41^b^	2,311 ± 0. 72^c^
Set back, (FV–PV) (RVU)	3,653 ± 0.02^a^	3169 ± 0.11^b^	1,076 ± 0.17^c^
Peak time, (min)	4.33 ± 0.25^a^	4.13 ± 0.62^a^	5.67 ± 0.11^b^
Pasting temperature, (ºC)	80.90 ± 0.11^a^	81.60 ± 0.18^a^	88.05 ± 0.16^b^
Gelatinization			
Onset temperature, *T*_o_ (ºC)	57.50 ± 0.58^c^	66.10 ± 0.74^a^	61.70 ± 0.72^b^
Peak temperature, *T*_p_ (ºC)	74.94 ± 0.61^b^	78.45 ± 0.71^a^	75.86 ± 0.87^a^
Conclusion temperature, *T*_c_ (°C)	92.00 ± 0.85^b^	93.70 ± 0.91^a^	89.93 ± 1.88^b^
Gelatinization temp. range, *T*_c_–*T*_o_ (°C)	34.50	27.60	28.23
Enthalpy change, ΔH (J/g)	5.57 ± 1.39^b^	9.56 ± 1.95^a^	4.97 ± 0.84^b^

Results are means ± standard deviation of triplicate determinations. Means followed by different superscript in the same row are significantly different (*p* < .05).

Breakdown viscosity which is the difference between the peak and trough viscosity was significantly reduced after hydrothermal modifications from 4252 RVU in BNS to 3925 RVU and 226RVU in BAS and BHS, respectively (Table 2). The relatively low value of breakdown viscosity of BHS could be an indication that heat‐moisture treated starch granules is susceptible to degradation during pasting with a concomitant decrease in the degree of crosslinking among the starch molecule (Hoover et al., 2010). The pasting temperature of the starch increased after modification in the following order: BHS > BAS > BNS. The increase in pasting temperature can be attributed to changes in structure, the increase of crystallinity, and the transition of the partial amorphous region to crystallinity after hydrothermal modification (Zavareze & Guerra Dias, 2011).

The gelatinization profile of the starches was enhanced by hydrothermal modification (Table 2). Hydrothermal modification increased the onset temperature (*T*_o_), peak temperature (*T*_p_), and enthalpy change (ΔH) of the BG starch. These increase showed that hydrothermal modification leads to the elevated thermal transition temperature, which is indicative of rearrangement of starch molecular chains to form molecular orders (helices/crystallites) with enhanced thermal stability (Wang, Zhang, Chen, & Li, 2016). The gelatinization range (*T*_c_–*T*_o_) of the starches was 27.60–34.50°C, this is similar to the 22.50–25.40°C reported for runner bean (Piecyk et al., 2013), but is lower than that reported for other leguminous starches such as black bean (62.5–82.0°C), pinto bean (59–82°C), field pea (54–9.0°C), and lentil (56–69°C) (Hoover & Manuel, 1996). The gelatinization range of the BG varied significantly in the following order: BNS > BHS > BAS. The presence of crystallites with different thermal stabilities inside the crystalline regions is responsible for the difference in gelatinization temperature range (Ovando‐Martinez, Osorio‐Diaz, Whitney, Bello‐Perez, & Simsek, 2011; Wang et al., 2017). The increase in onset gelatinization temperature (*T*_o_) after hydrothermal treatment from 57.50°C (BNS) to 66.10 and 61.70°C (BAS and BHS, respectively) could be attributed to the increased interaction between amylose/amylose and amylose/amylopectin (Chung, Liu, & Hoover, 2009). The observed increase in *T*_o_ after hydrothermal treatment is similar to that reported for other starches (Wang et al., 2017; Wang et al., 2017).

BAS had the highest ΔH value (9.56 J/g), onset and peak temperature (66.10 and 78.45°C, respectively) but with the lowest gelatinization range of 27.60°C. Other reports also showed that annealing of starch leads to increase in ΔH and gelatinization temperatures (*T*_o_, *T*_p_, *T*_c_), with a decrease in gelatinization temperature range (*T*_c_–*T*_o_) (Jayakody & Hoover, 2008). The increase in the gelatinization temperatures (*T*_o_, *T*_p_) of BAS could be attributed to the greater influence annealing has on *T*_o_ which represent the melting of the weakest crystallite. ΔH value of the starch decreased after heat‐moisture treatment from 5.57 J/g (BNS) to 4.97 J/g (BHS), this decrease could either be as a result of disturbance of the double helices present in the crystalline and non‐ crystalline regions of the granules (Gunaratne & Hoover, 2002) or as a result of partial gelatinization of amylose and amylopectin molecules that are less stable during heating.

### 3.4 Starch crystallinity

**Figure 5:**
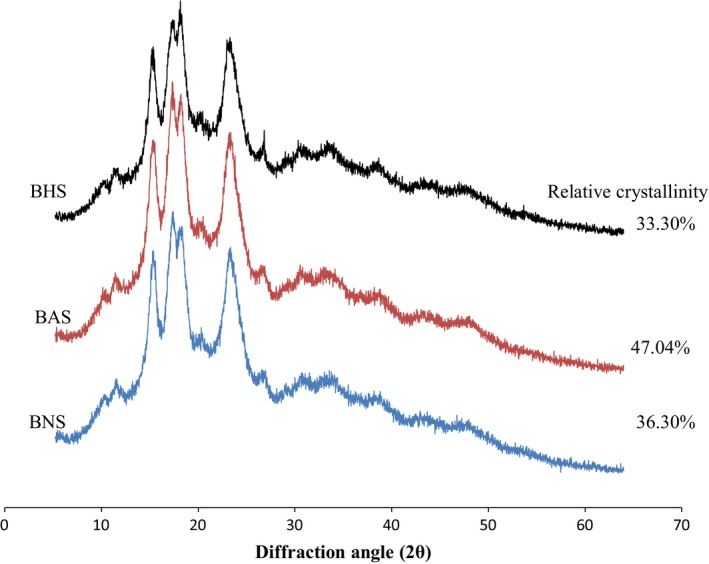
X‐ray diffractogram of native (BNS), annealed (BAS), and heat moisture treated (BHS) bambara groundnut starch

The native starch, BNS with strong peaks at 5.85 Å and 5.16 Å, medium peak at 3.85 Å and weak peak at 3.37 Å (Figure 5) exhibit “Type C” pattern which is characteristic of legume. This crystalline pattern was attributed to a mixture of “A” and “B” polymorphs (Afolabi, 2012). The native starch crystallinity pattern changed from “Type C” to “Type A” after heat‐moisture treatment (BHS) with a weak peak at 8.05 Å, medium peak at 3.88 Å and a strong peak at 5.87 Å and 5.17 Å. The presence of “Type A” crystallinity pattern in BHS is similar to reports on potato (Vermeylen, Goderis, & Delcour, 2006) and yam starch (Gunaratne & Hoover, 2002). According to Zavareze and Guerra Dias (2011), the effect of heat – moisture treatment on crystallinity depends on the source of the starch and treatment conditions. Annealing did not alter the crystallinity of the starch, BAS exhibit the “Type C” diffractogram with a weak peak at 7.95 Å and 3.40 Å, medium peak at 3.87 Å and a strong peak at 5.17 Å. This indicates that the expected change in orientation of starch crystallites, crystallite perfection, and formation of amylose crystallites may have been in low magnitude after annealing (Jacobs & Delcour, 1998).

The relative crystallinity of BNS, BAS, and BHS was 36.30%, 47.04%, and 33.77%, respectively (Figure 5), this is higher than the 17–25% range reported for several pulses (Hoover & Ratnayake, 2002). The decrease in starch's crystallinity from 36.30% (BNS) to 33.77% (BHS) is indicative of partial disruption of the starch crystallites due to heat‐moisture treatment. This observation is in agreement with reported decrease in starch crystallinity after heat‐moisture treatment (Wang et al., 2016).

### 3.5 Granule morphology

**Figure 6:**
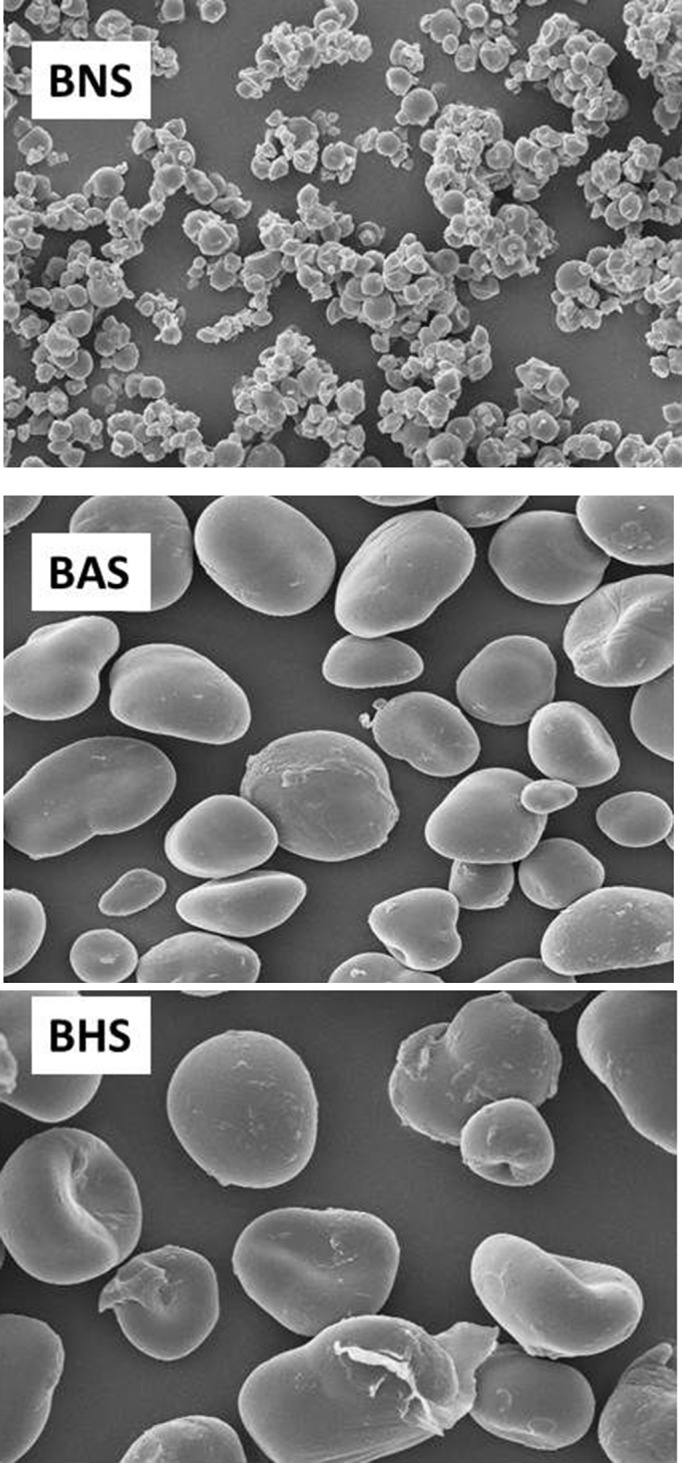
The scanning electron micrograph of the native (BNS), annealed (BAS), and heat moisture treated (BHS) bambara groundnut starch at 500X magnification

The native BG starch granule was oval, with few mild rupture, and diameter is between 22 and 30 μm (Figure 6), this is within the range of 10–45 μm reported by Jane, Kasemsuwan, Leas, Zobel, and Robyt (1994) for pulses with oval granule morphology. The presence of the mild rupture could be attributed to the isolation method of the starch (Adebowale et al., 2005). Annealing and heat‐moisture treatment of the starch did not have any significant effect on the granule size, surface morphology, and granule size distribution; this observation is in tandem with reports on starches like oat, wheat, barley, lentil, finger millet, and potatoes (Adebowale et al., 2005; Jacobs & Delcour, 1998; Wang et al., 2017).

### 3.6 Starch digestibility

**Figure 7:**
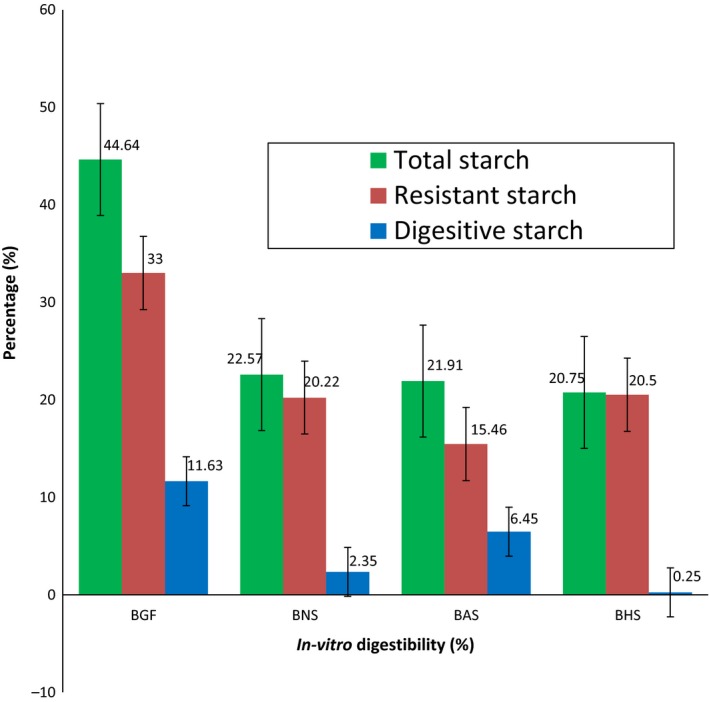
The total starch, resistant starch, and digestive starch content of Bambara groundnut; flour (BGF), native starch (BNS), annealed starch (BAS), and heat moisture treated starch (BHS)

The in‐vitro digestibility of BG flour, native (BNS), heat moisture treated (BHS), and annealed (BAS) starches was presented in Figure 7. The 33% resistant starch of BG flour was lower than the 35.00% reported for red kidney (Eyaru, Shrestha, & Arcot, 2009), but higher than the 16.43% reported for chickpea (Garcia‐Alonso, Goni, & Saura‐Calixto, 1998), 25.40% for lentil (Bednar et al., 2001), and 11.03% for faba bean flours (Ambigaipalan et al., 2011). However, the 44.64% total starch content of the BG flour is within the 22%–45% (Utrilla‐Coello et al., 2014) and 33%–88% (Morales‐Medina, Munio, Guadix, & Guadix, 2014) reported for different leguminous flours.

The resistant starch content of the native, heat moisture treated, and annealed BG starch increased in the following order: BHS > BNS > BAS (Figure 7). Therefore, heat moisture treatment increased the resistant starch content of the BG starch. Increase in resistant starch content after heat‐moisture treatment was reported for several starches (Huang, Zhou, Jin, Xu, & Chen, 2016; Hung, Vien, & Phi, 2016; Teixeira et al., 2016). There was little or no correlation between the resistant starch and amylose content of the BG starches since the later increased in the following order: BHS > BAS > BNS (Table 1). This poor correlation between the resistant starch and amylose content was also reported for starches isolated from different botanical sources (Vasanthan & Bhatty, 1998; Walter, da Silva, & Denardin, 2005; Zhang et al., 2007). Hence, the formation of resistant starches may be attributed to the molecular association between starch components, the degree of crystallinity, and starch gelatinization properties (Zhang et al., 2007). The increase in the resistant starch content of BHS may also be due to some interactions formed during the treatment which may have survived after gelatinization, thereby partly restricting accessibility of starch chains to the hydrolyzing enzymes (Hung et al., 2016). This is corroborated by the enthalpy change which decreased after heat moisture treatment of the starch (Table 2).

The digestible starch content of the BG starch increased in the following order: BAS > BNS > BHS (Figure 7). The above observation is consistent with the starch's degree of crystallinity and enthalpy of gelatinization (ΔH) which also increased in the following order BAS > BNS > BHS (Table 2). However, the resistant starch content of the BG starch increased in the reverse order BHS > BNS > BAS. This is an indication that any treatment or conditions that increased the resistant starch content of the starch may ultimately reduce its digestible starch (and vice versa). Hydrothermal modification of the BG starch also leads to decrease in the total starch content of the native starch in the following order: BNS > BAS > BHS (Figure 7).

## 4 CONCLUSION

BG flour and starch is an excellent source of resistant starch, and the versatility of its starch can be enhanced by heat‐moisture treatment and annealing. The use of BG flour and heat‐moisture treated starch in food and food products may be an excellent way to alleviate obesity and diabetes, due to its high resistant starch content. Heat‐moisture treatment also reduced the amylose content of the starch with a consequential decrease in its swelling power, this is a desirable property in the application of the modified starch in food and allied industries.

## CONFLICT OF INTEREST

The authors hereby state that there is no conflict of interest to declare on this research.
